# Safety of intracameral application of moxifloxacin and dexamethasone (Vigadexa®) after phacoemulsification surgery

**DOI:** 10.1007/s00417-023-06095-0

**Published:** 2023-05-25

**Authors:** Virgilio Galvis, Angelica Maria Prada, Alejandro Tello, Maria Margarita Parra, Paul Anthony Camacho, María Paz Polit

**Affiliations:** 1 Centro Oftalmológico Virgilio Galvis, Calle 158 20–95, Consultorio 301, Torre C, Cañaveral, Floridablanca, Santander Colombia; 2 Universidad Autónoma de Bucaramanga (UNAB), Bucaramanga, Colombia; 3 Fundación Oftalmológica de Santander (FOSCAL), Floridablanca, Colombia; 4 Universidad Industrial de Santander (UIS), Bucaramanga, Colombia

**Keywords:** Cataract, Phacoemulsification, Endothelial cell count, Endothelial cell loss, Prophylaxis of endophthalmitis

## Abstract

**Background:**

Intracameral antibiotics, such as moxifloxacin and cefuroxime, are safe to corneal endothelial cells and effective prophylaxis of endophthalmitis after cataract surgery. Corneal endothelial cells decrease in density after cataract surgery. Any substance used in the anterior chamber may affect corneal endothelial cells and lead to a greater decrease in density. This study wants to determine the percentage of endothelial cell loss after cataract extraction by phacoemulsification with off-label intracameral injection of moxifloxacin and dexamethasone (Vigadexa®).

**Methods:**

An observational retrospective study was performed. The clinical records of patients undergoing cataract surgery by phacoemulsification plus intracameral injection of Vigadexa® were analyzed. Endothelial cell loss (ECL) was calculated using preoperative and postoperative endothelial cell density. The relation of endothelial cell loss with cataract grade using LOCS III classification, total surgery time, total ultrasound time, total longitudinal power time, total torsional amplitude time, total aspiration time, estimated fluid usage, and cumulative dissipated energy (CDE) was studied using univariate linear regression analysis and logistic regression analysis.

**Results:**

The median loss of corneal endothelial cells was 4.6%, interquartile range 0 to 10.4%. Nuclear color and CDE were associated with increased ECL. ECL>10% was associated with age and total ultrasound time in seconds.

**Conclusions:**

The endothelial cell loss after the intracameral use of Vigadexa® at the end of cataract surgery was similar to the reported in other studies of cataract surgery without the use of intracameral prophylaxis for postoperative endophthalmitis (POE). This study confirmed the association of CDE and nuclear opalescence grade with postoperative corneal endothelial cell loss.


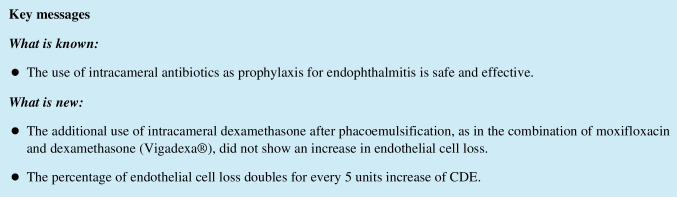


## Introduction

Cataract affects 94 million people worldwide and is the most prevalent cause of vision impairment and blindness [1]. Cataract surgery rates continue to grow globally, and in some areas of the world at a rapid rate. In Latin America they increased by 47% from 2008 to 2013, reaching 2,672 surgeries per 1 million inhabitants [2]. Postoperative endophthalmitis (POE) is a severe complication of cataract surgery. Rangel et al. reported phthisis bulbi in 5.7% endophthalmitis cases related to intraocular surgery [3].

The European Society of Cataract and Refractive Surgeons (ESCRS) found that intracameral injection of cefuroxime at the end of cataract surgery reduced the incidence of endophthalmitis fivefold [4]. Given the evident difference found while conducting the study, the Data Monitoring Committee Monitoring advised that it would be unethical to finish the clinical trial as planned, and recruitment was closed early [4]. Melega et al. found a significant reduction in the incidence of endophthalmitis in patients treated with moxifloxacin at the end of cataract surgery compared with those without intracameral antibiotic, 0.05% and 0.38%, respectively [5]. A recent survey among members of the American Society of Cataract and Refractive Surgery (ASCRS) found a rise in cataract surgeons using intracameral antibiotic prophylaxis compared to 2007 [6]. The use of intracameral antibiotics at the conclusion of cataract surgery was more frequent among European respondents than among US respondents, 70% vs 30%, respectively [6]. The 2022 American Academy of Ophthalmology (AAO) Cataract Preferred Practice Pattern Guidelines and the 2013 ESCRS guidelines for prevention and treatment of endophthalmitis [7] agree that there is strong evidence supporting the safety and efficacy of the intracameral injection of antibiotic as prophylaxis for POE. A recent systematic review and meta-analysis confirmed the benefit of using intracameral antibiotics at the end of cataract surgery in reducing POE rates [8]. Given the evidence, it is our belief that intracameral antibiotic at the conclusion of cataract surgery is the best practice for the prophylaxis of POE.

On another note, the intraocular use of steroids, such as dexamethasone and triamcinolone, has proven to be safe and effective in managing intraocular inflammation [9–12]. Intraocular steroids can be used as intravitreal injections [13], trans-zonular injections [14], injections into the anterior chamber [15], or with delayed release intravitreal devices [16].

One of the risks that arise when using substances injected in the anterior chamber is the toxicity to the human corneal endothelial cells (HCEC). HCEC have mitosis almost completely inhibited in vivo [17]. Therefore, if the ECL is significant and the postoperative density is very low, secondary irreversible corneal edema (pseudophakic bullous keratopathy, PBK) may present and a corneal transplant may be required. PBK is the main cause of keratoplasty after cataract surgery [18–20]. Toxicity to HCEC and toxic anterior segment syndrome (TASS) are potential concerns when using intraocular substances. Toxic anterior segment syndrome (TASS) is a severe sterile inflammatory complication of cataract surgery [21] After TASS, HCEC have been reported to have less density, higher coefficient of variation, and less hexagonality [21].

It is therefore very important that the substances injected intraocularly have no harmful effects on corneal endothelial cells. Experimentally, it has been shown that intracamerally injected preservative-free 0.5% moxifloxacin/0.1% dexamethasone ophthalmic solution (Vigadexa, Alcon, Fort Worth, TX, USA) was safe on HCEC in an in vivo rabbit model [22]. This agrees with other in vitro studies, which used moxifloxacin (Vigamox®) in cultured HCECs [23–25]. However, other researchers have found a dose-dependent damage to HCECs [26]. On the other hand, intracameral moxifloxacin (both off-label Vigamox® and formulations made for intraocular use) has been extensively used in humans, and no significant ECL has been reported [26–28].

Therefore, the idea of adding the anti-inflammatory effect of dexamethasone to the prophylactic antibiotic seems to be very reasonable. The objective of the present study was to evaluate the ECL in eyes that received an off-label intracameral injection of Vigadexa® as prophylaxis for POE at the end of cataract surgery.

## Methods

A retrospective observational study was performed. The clinical records of patients undergoing cataract extraction surgery by coaxial microincision phacoemulsification from January to June 2016 were evaluated. All surgeries were performed by a single surgeon (VG) using the Centurion Vision System ™ with the Intrepid® Balanced tip (Alcon). To be eligible for inclusion, patients must have had an intracameral injection of Vigadexa® at the end of surgery. Eyes were excluded from the study if there were records of previous intraocular surgery, combined glaucoma and cataract surgery, uveitis, and/or corneal endothelial dystrophy. Also, eyes with intraoperative complications, requiring the use of adrenaline in the anterior chamber or the use of iris retractor hooks, were excluded. Study protocol was approved by the ethical committee at Fundación Oftalmologica de Santander (FOSCAL). All procedures performed involving human participants were in accordance with the ethical standards at FOSCAL clinic and with the 1964 Helsinki Declaration and its later amendments or comparable ethical standards. All patients gave written consent for the use of their clinical data for research and publication purposes.

HCEC were evaluated using a non-contact specular microscope (SP3000P, Topcon, Tokyo, Japan). Preoperative and 3-month postoperative endothelial cell density, coefficient of variation, percentage of hexagonal cells, and mean size were collected. The percentage of ECL was defined as [(preoperative endothelial cell density − postoperative endothelial cell density)/preoperative endothelial density] × 100.

Lens opacity classification system III (LOCS III) was used to grade all cataracts. The LOCS III evaluates four aspects of the crystalline lens, nuclear opalescence (NO), nuclear color (NC), cortical opacity (C), and posterior subcapsular opacity (P). The grades are in the decimal scale, NO and NC from 0.1 to 6.9, and C and P from 0.1 to 5.9. The procedure to grade the cataract has already been described [29]. All cataracts were graded by one surgeon. The parameters used during the surgery were adjusted according to the classification of the cataract. Cataracts graded NO 0 to 4.9, NC 0 to 4.9, P 0 to 5, and C 0 to 5 were considered to be soft to medium in hardness. For such cataracts, the parameters used were flow/aspiration rate of 25 to 50 cm^3^/min, torsional amplitude between 20 and 50%, and vacuum from 100 to 650 mm Hg. Cataracts graded NO 5 or more and NC 5 or more were considered hard. For such hard cataracts, the torsional amplitude was increased to a range between 30 and 70%, without change in flow/aspiration rate or vacuum.

All cataract surgeries were performed with the “divide and conquer” technique. The combination of 3% sodium hyaluronate and 4% chondroitin sulfate (Viscoat®, Alcon, Forth Worth, TX, USA) was used in all surgeries. At the end of the procedure, undiluted Vigadexa® 0.05ml (moxifloxacin 0.5% and dexamethasone 0.1%) sterile ophthalmic solution was administered intracamerally. Such volume corresponds to 250 μg of moxifloxacin and 50 μg of dexamethasone. The intraocular use of Vigadexa® is off-label. Patients also received standard postoperative treatment with topical moxifloxacin 0.5% one drop every 3 h for 1 week and prednisolone 1% 1 drop four times a day and tapering off over a period of 4 weeks.

Preoperative ocular biometry data was collected from clinical records. Ocular biometry data included axial length (AL), lens thickness (LT), and anterior chamber depth (ACD). For ocular biometry, an optical biometry was the standard exam (Lenstar, Haag Streit), in eyes where opacity prevented the performance of the optical biometer, and ultrasound biometry was the alternative exam. Intraoperative parameters from the Centurion® device were obtained, which included total surgery time, total ultrasound time, total longitudinal power time, total torsional amplitude time, total aspiration time, estimated fluid usage, and cumulative dissipated energy (CDE).

The data was entered into an Excel sheet (Microsoft Excel, Microsoft Corporation, Redmond, WA, USA). STATA program (StataCorp, College Station, TX, USA) was used for analysis. Each patient eye was given a code to pseudonymize the data. The variables were analyzed by descriptive statistics, estimating the measures of central tendency and dispersion. Normality was evaluated using the Shapiro–Wilk test. The mean and standard deviation are reported unless specified otherwise. Differences between pre- and post-endothelial cell characteristics were evaluated using the Wilcoxon signed-rank test. Univariate and multivariate lineal regression analysis was done with ECL as the outcome measure. Logistic univariate and multivariate regression analysis was used to estimate the odds of ECL>10%. ECL of 10% is the value of the third quartile of ECL in the sample, so it was used as the cut off for the dichotomized ECL. A stepwise multivariable analysis was performed. Missing data was handled using pairwise deletion. A two-sided *P* <0.05 was considered statistically significant.

### Sample size calculation

The normal decrease in endothelial cell count annually is around 0.53% [30]. ECL after cataract surgery with phacoemulsification has been reported to be 11.6±14.3% [28]. Thus, to detect a mean difference between pre- and postoperative endothelial cell count of 11.6% with 80% power using a 5% level two-sided test, the sample size was calculated to be 23 eyes.

## Results

A total of 159 eyes of 101 patients were included in the study; 46.5% were men, and 50.3% were right eyes. The average age was 67.4 ± 10.3 years (34–87 years). Preoperative LOCS III classification, biometric measurements, and intraoperative parameters are presented in Table 1. Data for LOCS III classification was available for all eyes. Ocular biometry data was measured using an optical biometer (Lenstar, Haag Streit) in 154 (96,8%), and in 5 eyes, the opacity of the cataract prevented the use of the optical biometer; in which case, the ultrasound biometer was used (OcuScan, Alcon). There were missing values for LT, ACD, torsional amplitude time, longitudinal power time, total ultrasound time, and total surgery time. The percentage of missing data across the variables varied between 1.8% (*n*=156 for ACD) and 9.4% (*n*=134 for LT). Table 1 provides the number of eyes with data available for each variable.

**Table 1:** Preoperative and intraoperative parameters

		*n*	Mean ± SD
Age		159	67.4 ± 10.3
Sex			
Male		75	47.1%
Female		84	52.8%
Eye			
Right		80	50.3%
Left		79	49.6%
Cataract classification (LOCS III)	NO	159	2.7 ± 1.3
	NC	159	2.6 ± 1.3
	P*	159	0 (0–2)
	C*	159	0 (0–1)
AL (mm)*		159	23.34 (22.46–23.98)
ACD (mm)		156	3.13 ± 0.41
LT (mm)		134	4.48 ± 0.42
Torsional amplitude time (seconds)*		154	16.2 (8.7–29.7)
Longitudinal power time (seconds)*		155	0 (0–0.1)
Total ultrasound time (seconds)*		155	16.4 (9.0–30.50)
Aspiration time (minutes)*		159	0.31 (0.25–0.37)
Total surgery time (minutes)*		155	11.50 (9.14–15.36)
CDE*		159	2.83 (1.59–5.99)
Estimated fluid usage (ml)*		159	133 (117–160)

*AL* axial length, *ACD* anterior chamber depth, *LT* lens thickness, *CDE* cumulative dissipated energy

*median (interquartile range)

Changes in parameters of corneal endothelium are shown in Table 2. Data of endothelial cell parameters were available for all eyes (*N*=159). The average endothelial cell density diminished from 2339.9 ± 384.0 cells/mm^2^ before the procedure to 2195.1 ± 437.5 cells/mm^2^ 3 months after the surgery (*p*<0.001). The median loss of corneal endothelial cells was 4.6%, with an interquartile range (IQR) from 0 to 10.4%. Also, 22.1% (35 eyes) showed higher values of endothelial cell density after surgery (range 0.03–12.5%). No patient had signs of a toxic anterior segment syndrome (TASS) after cataract surgery.

**Table 2:** Endothelial cell density before and 3 months after surgery

	Preoperative	Postoperative	Change after surgery	*p*
Cell density (cells/mm^2^) ^‡^	2339 ± 384.09	2195.14 ± 437.54	−98.40 (−255–0) †	<0.001
Percentage of hexagonal cells (%)^‡^	51.65% ± 8.29%	49.60% ± 9.80%	−1% (−2–5%) †	0.001
Size (μm^2^) ^‡^	429.50 (393.10–485.60) †	450.20 (403.20–510.30) †	13.1 (0–33.5) †	<0.001
Coefficient of variation (%)^‡^	37.20% (34.40–41.30%) †	37.30% (34.50–41.10%) †	0 (1.7–2.2%) †	0.348

*Wilcoxon sign rank test for difference between pre- and post-endothelial cell variables

†Non-normal distribution by Shapiro–Wilk test. Median (interquartile range)

‡Analysis was done with the full data set *N*=159

### Endothelial cell density change analysis

After univariate linear regression analysis, the following variables were positively correlated with an increased risk of ECL: age, NO and NC LOCSIII cataract classification, torsional amplitude time, longitudinal power time, ultrasound total time, aspiration time, CDE, and estimated fluid usage. After multivariate backwards analysis, NC grade (beta coefficient 0.021, *p*=0.004) and CDE (beta coefficient 0.005, *p*=0.016) were associated with increased ECL. In addition, CDE was categorized in increases of 5 units, as follows: group 0 with CDE<5; group 1 with CDE higher than 5 and lower or equal to 10; group 2 with CDE higher than 10 and lower or equal to 15; and group 3 with CDE higher than 15. The difference of ECL in cases with the same NC grade but with different CDE was 3.9% (*p*<0.05) between group 0 and 1; 7.3% (*p*<0.05) between group 0 and 2; and 15.0% between group 0 and 3.

### Percentage of ECL >10%

After univariate logistic regression analysis, the following variables were positively correlated with an increased risk of ECL >10%: age, NO and NC grades, torsional amplitude time, longitudinal power time, ultrasound total time, aspiration time, and CDE. After multivariate backwards analysis, age (OR 1.04; *p*=0.014) and ultrasound total time in seconds (OR 1.03; *p*=0.001) were associated with increased risk of ECL >10%.

## Discussion

The present study found the median ECL to be 4.6% (IQR 0% to10.4%), which is in the range of that reported in studies on phacoemulsification with intracameral antibiotics (between 3.6% and 18.3%) (Table 3) [28, 31, 32]. The wide range of ECL reported in the literature may be explained by the fact that ECL is influenced by many different factors, including hardness of the cataract (in turn related to the amount of ultrasonic energy applied), the technology used during surgery (longitudinal phaco, torsional phaco, previous division of the nucleus with manual instruments or femtosecond laser, etc.), the experience of the surgeon, and the surgical technique (divide and conquer, phaco chop, double chop) [33–41].

**Table 3:** Recent selected studies on postoperative corneal endothelial cell loss following phacoemulsification with intracameral antibiotics

Authors	# of eyes	Follow-up time after surgery (weeks)	Intracameral substance	Preoperative ECD		Postoperative ECD		Mean ECL (%)
				Mean cells/mm^2^	SD	Mean cells/mm^2^	SD	
Pérez-Canales et al. (2015) [31]	26	1	Vancomycin 1 mg/0.1 ml	2289.12	394.75	1957.1	443.48	14.5
Pérez-Canales et al. (2015) [31]	26	4	Vancomycin 1 mg/0.1 ml	2289.12	394.75	1952.27	395.74	14.7
Pérez-Canales et al. (2015) [31]	26	12	Vancomycin 1 mg/0.1 ml	2289.12	394.75	1984.58	417.25	13.3
Pérez-Canales et al. (2015) [31]	26	1	Cefuroxime 1 mg/0.1 ml	2441.31	350.64	2154.88	462.66	11.7
Pérez-Canales et al. (2015) [31]	26	4	Cefuroxime 1 mg/0.1 ml	2441.31	350.64	2121.35	443.84	13.1
Pérez-Canales et al. (2015) [31]	26	12	Cefuroxime 1 mg/0.1 ml	2441.31	350.64	2147.04	464.51	12.1
Lucena et al. (2018) [32]	1016	4	Moxifloxacin 150 μg/0.03 ml	2426	325	2177	283	10.3
Khalili et al. (2020) [33]	113	48	Moxifloxacin 250 μg/0.1 ml	3443	N/A	3320	N/A	3.6
Chang et al. (2020) [29]	25	4	Moxifloxacin 500 μg/0.1 ml	2700	N/A	2334	N/A	13.6
Chang et al. (2020) [29]	25	12	Moxifloxacin 500 μg/0.1 ml	2700	N/A	2387	N/A	11.6
Chang et al. (2020) [29]	25	4	Moxifloxacin 250 μg/0.1 ml	2721	N/A	2466	N/A	9.4
Chang et al. (2020) [29]	25	12	Moxifloxacin 250 μg/0.1 ml	2721	N/A	2496	N/A	8.3
Soro-Martínez et al. (2021) [34]	20	4	Cefuroxime 100μg/0.1 ml	2528	383	2066	434	18.3
Present study	159	12	Moxifloxacin 250 μg/0.05 ml + Dexamethasone 50 μg/0.05 ml	2339	384	2195	437	6.2

*ECD* endothelial cell density, *ECL* endothelial cell loss

The use of an intracameral substance (either an antibiotic or an antibiotic plus steroid) is considered a potential factor related to ECL. In this matter, this study analyzed the safety of the use of intracameral Vigadexa® after cataract surgery performed with only divide and conquer technique by a single surgeon and showed no differences in ECL with previously published literature (Table 3) [28, 31, 32, 42].

To our knowledge, there are no published articles regarding the safety of intracameral Vigadexa® as prophylaxis for POE after cataract surgery. Nonetheless, Aguilera and Escaf have presented their results at the 2012 ESCRS meeting in Milan (Aguilera F, Escaf L. Evaluation of ocular safety of prophylactic intracameral moxifloxacin-dexamethasone combination in cataract surgery, ESCRS Annual Congress 2012). Aguilera et al. showed that the combination of moxifloxacin and dexamethasone was safe, without anterior or posterior segment complications. Furthermore, they found that there was no statistical difference in ECL after cataract surgery either with the use of Vigadexa® or (balanced saline solution) BSS.

On another note, the present study found that for every increase in CDE of 1 unit, there was a 0.5% increase in ECL. Additionally, the percentage of ECL doubled with every 5-unit increase in CDE. ECL >10% was associated with age and total ultrasound time during surgery. Mahdy et al. also found a positive correlation between CDE and total ultrasound time with ECL [33].

Regarding the assessment of HCEC, non-contact specular microscope has been shown to be a reliable method [43]. In a Colombian cohort, the mean difference of endothelial cell density repeated measurements was 8.3 ± 168.9 cell/mm^2^ (i.e., 0.3% ± 6.1%) and 95% limits of agreement from −322.6 to 339.5 (i.e., −11.7% to 12.3%) [44]. The variability in repeated measurements may explain the increase in endothelial cell density in 22.1% eyes in the present study.

The limitations of the present study include the absence of a control group and the assessment of HCEC with only one image from the non-contact specular microscope, which could be related to the result of a no plausible increase of endothelial cell density in 22.1% of cases.

In conclusion, in the present study, the intracameral injection of a preservative-free combination of moxifloxacin and dexamethasone (Vigadexa®) at the end of cataract surgery did not show deleterious effects on the corneal endothelium at 3 months. Further studies are warranted to confirm the possible anti-inflammatory effects of intracameral dexamethasone in the early postoperative period after cataract surgery. Also, future studies should include a control group with the use of only an intracameral antibiotic.
